# Antenatal HIV testing in rural eastern Uganda in 2003: incomplete rollout of the prevention of mother-to-child transmission of HIV programme?

**DOI:** 10.1186/1472-698X-6-6

**Published:** 2006-05-03

**Authors:** Charles AS Karamagi, James K Tumwine, Thorkild Tylleskar, Kristian Heggenhougen

**Affiliations:** 1 Department of Paediatrics and Child Health, Makerere University, P.O.Box 7072, Kampala, Uganda; 2 Clinical Epidemiology Unit, Makerere University, P.O.Box 7072, Kampala, Uganda; 3 Centre for International Health, University of Bergen, Armauer Hansen Bldg, N-5021 Bergen, Norway; 4 Department of International Health, Boston University School of Public Health, 715 Albany Street, T4W, Boston, MA 02118, USA

## Abstract

**Background:**

Uganda began to implement the prevention of mother-to-child transmission (PMTCT) of HIV programme in 2000, and by the end of 2003 it had expanded to cover 38 of the 56 districts including Mbale District. However, reports from Mbale Hospital showed that less than 10% of pregnant women accepted antenatal HIV testing. We therefore conducted a study to determine the proportion of pregnant women who tested for HIV and the gaps and barriers in PMTCT implementation.

**Methods:**

The study was a cross sectional household survey of women aged 18 years or more, with children aged one year or less, who resided in Mbale Town or in the surrounding Bungokho County. We also conducted in-depth interviews with six health workers in Mbale Hospital.

**Results:**

In 2003, we interviewed 457 women with a median age of 24 years. The prevalence of antenatal HIV testing was 10 percent. The barriers to antenatal HIV testing were unavailability of voluntary counselling and testing services (44%), lack of HIV counselling (42%) and perceived lack of benefits for HIV infected women and their infants. Primipara (OR 2.6, 95% CI 1.2–5.8), urban dwellers (OR 2.7, 95% CI 1.3–5.8), women having been counselled on HIV (OR 6.2, 95% CI 2.9–13.2), and women with husbands being their primary confidant (OR 2.3, 95% CI 1.0–5.5) were independently associated with HIV testing.

**Conclusion:**

The major barriers to PMTCT implementation were unavailability of PMTCT services, particularly in rural clinics, and poor antenatal counselling and HIV testing services. We recommend that the focus of the prevention of mother-to-child transmission of HIV programme should shift to the district and sub-district levels, strengthen community mobilization, improve the quality of antenatal voluntary counselling and HIV testing services, use professional and peer counsellors to augment HIV counselling, and ensure follow-up care and support for HIV positive women and their infants.

## Background

It is estimated that 2.1 million children are infected with HIV worldwide. About 90% of the HIV-infected children are in Africa and over 95% of HIV infections in children below the age of 15 years are due to mother-to-child-transmission[1]. Antenatal voluntary counselling and HIV testing (VCT), followed by the provision of short-course nevirapine prophylaxis is the key intervention advocated in the prevention of mother-to-child-transmission of HIV (PMTCT)[2,3]. VCT acceptance by pregnant women varies greatly and is influenced by several factors including fear of disclosure of HIV results, stigma, discrimination, disempowerment, fatalism, accessibility of VCT services, or perceived lack of benefits [3-8].

The Uganda Ministry of Health's policy on the prevention of mother-to-child transmission of HIV states that all pregnant women should have access to free voluntary counselling and HIV testing, counselling on infant feeding, and intrapartum and postnatal short-course nevirapine prophylaxis. Uganda began to implement the PMTCT programme in the year 2000, and by the end of 2003 it had expanded to cover sites in 38 of the 56 districts including Mbale District[9]. The Ministry of Health is responsible for implementing the policy in the regional (e.g. Mbale Hospital) and district hospitals while each district is responsible for implementing the policy in the health centres and rural clinics at the health sub-district. However, reports from Mbale Hospital in 2003 showed that less than 10% of pregnant women accepted antenatal HIV testing. During November and December 2003, we conducted this study to determine: (a) the proportion of women who tested for HIV; (b) the gaps in the PMTCT implementation; and (c) the barriers to PMTCT implementation during the most recent pregnancy.

## Methods

Mbale District is situated in Uganda's eastern region and is divided into 4 counties namely Bubulo, Bunghoko, Manjiya, and Mbale Town. It had a population of over 720 000 in 2003, of which 90% was, like the rest of Uganda, rural. The population was predominantly *Bagisu *and the main language was *Lumasaba*. The literacy rate was 64% for men and 49% for women, and the main economic activity was subsistence farming[10]. In 2003, the HIV prevalence was reported to be 5.6%[11].

The study was a cross sectional household survey of women with infants, combined with in-depth interviews of health workers. Mbale Town and the surrounding Bungokho County were purposively selected to provide a rural and an urban sample. We used the WHO/EPI cluster survey method and defined a cluster as a village (rural) or ward (town). We randomly selected 68 villages/wards, each consisting of about 300 households. We identified the centre of each cluster where we spun a bottle on the ground to determine the direction of the interviewers. We randomly selected the starting point (household) from the households between the centre and boundary of the cluster. The second household was the one nearest the starting point and the third household was the one nearest the second household. At the boundary of the cluster, the interviewers turned clockwise and continued to select households until a total of seven households were selected in the same way. Only the households that fulfilled the selection criteria were selected.

Women aged 18 years or more, with children aged one year or less, who resided in the selected households in Mbale Town or Bungokho County were recruited into the study and after consent, they were interviewed. The interviewer-administered questionnaire used contained 66 items divided into 3 sections on 1) socio-demographic and socio-economic characteristics of the woman and her husband, including radio ownership and access to health services in terms of the household's distance from available PMTCT services (> 5 kms = poor access), 2) antenatal and postnatal experience related to the youngest child, and 3) perceptions and actions related to antenatal VCT, infant feeding counselling, and intrapartum/postnatal nevirapine prophylaxis. The questionnaire was pretested by the investigators and twelve trained research assistants who were fluent in English and the local language (*Lumasaba*). The research assistants worked in six pairs of a woman and a man and administered all the items of the questionnaire to all women in the study.

We based our sample size calculation on estimation of the prevalence of HIV testing in pregnant women in Mbale District. We used an expected proportion of HIV tested women of 0.15, a total width of 0.10, a confidence level of 95%, and a design effect of 2.0 because of the cluster design. We increased the sample size by 20% to cater for problems that might occur in recruitment. The estimated sample size was 476, but since 19 women were not available for interview, and we failed to get replacements for them, the final sample size was 457 women. There were no refusals. Quantitative data was entered into EPINFO version 6.04 and then exported to Stata version 8.0 for analysis that adjusted for the design effect. Bivariate analysis was performed between HIV test of the woman as the dependent variable and each independent variable. Variables that were significant at the level of P < 0.2 were then entered into a model for logistic regression to control for confounding.

We conducted in-depth interviews with six health workers involved in the PMTCT programme at Mbale Hospital. The health workers were selected purposively because they were the staff responsible for the PMTCT services. The interviews were conducted in English and each participant was given a fictitious name that was used when analysing the data. The interviews were semi-structured to allow for sensitive issues to be discussed freely. The interviews lasted between one to two hours and were tape-recorded, transcribed and coded. The data was examined for emerging and recurrent themes and views that were in agreement or opposition to the themes.

Institutional permission to conduct the study was obtained from Makerere University Faculty of Medicine Research and Ethics Committee and informed consent was obtained from all participants in the study.

## Results

### Socio-demographic characteristics

Of the 457 women enrolled in the study, three quarters were living in rural villages and had a median age of 24 years (range 15 – 45 years; Table 1). Most women were Muslim, worked in agriculture, were in a consensual relationship and had less than 8 years of education. The husbands were older than their wives/partners with a median age of 30 years (range 19 – 81 years), had similar educational levels as the women and also worked mainly in agriculture.

**Table 1:** Frequency distribution and logistic regression of factors influencing HIV testing of 457 women, Mbale, Uganda

Variable	Tested n (%)	Did not test n (%)	Unadjusted OR (95% CI)	Adjusted OR (95% CI)
Residence				
Rural	24(07)	309(93)	1.0	
Urban	21(17)	103(83)	2.6(1.4 – 4.9)	2.7(1.3 – 5.8)

Age of mother				
15 – 24 years	24(10)	214(90)	1.0	
25 – 45 years	21(10)	198(90)	0.9(0.5 – 1.8)	

Education of mother				
0 – 7 years	24(07)	300(93)	1.0	
8 years or more	21(16)	112(84)	2.3(1.3 – 4.4)	

Occupation of mother				
Agriculture	36(09)	364(91)	1.0	
Other	9(16)	48(84)	1.9(0.9 – 4.2)	

Radio ownership				
No	6(06)	97(94)	1.0	
Yes	35(11)	285(89)	2.0(0.8 – 4.9)	

Religion of mother				
Muslim	23(08)	254(92)	1.0	
Christian	22(12)	158(88)	1.5(0.8 – 2.9)	

Marital status				
Married/cohabiting	39(09)	379(91)	1.0	
Single/widowed	6(15)	33(85)	1.8(0.7 – 4.5)	

Parity				
Multipara	25(07)	332(93)	1.0	
Primipara	20(20)	80(80)	3.3(1.8 – 6.3)	2.6(1.2 – 5.8)

Delivery in a health facility?				
No	14(06)	227(94)	1.0	
Yes	31(14)	185(86)	2.7(1.4 – 5.3)	

Who is your primary confidant?				
Another woman	27(09)	272(91)	1.0	
Husband	10(17)	50(83)	2.0(0.9 – 4.4)	2.3(1.0 – 5.5)

Age of husband				
18 – 24 years	4(06)	65(94)	1.0	
25 years or more	31(12)	229(88)	2.2(0.7 – 6.5)	

Education of husband				
0 – 7 years	14(07)	198(93)	1.0	
8 years or more	21(13)	139(87)	2.1(1.1 – 4.3)	

Do you fear to test for HIV?				
No	40(11)	335(89)	1.0	
Yes	5(06)	76(94)	0.6(0.2 – 1.4)	

HIV counselling				
No	17(05)	325(95)	1.0	
Yes	28(24)	87(76)	6.5(3.4–12.5)	6.2(2.9–13.2)

### Gaps in PMTCT implementation

In order for the PMTCT programme to be 100% effective, all mothers need to attend an antenatal clinic, be counselled, test for HIV and deliver in a health unit. At the time of the survey, only Mbale Hospital within the study area provided PMTCT services including antenatal VCT. Thus women who attended antenatal services at other health units could not access antenatal VCT services. However, even some of the women attending Mbale Hospital were not counselled even though the PMTCT services were available in the hospital. Furthermore, there were women who were tested for HIV outside the antenatal setting but without having been counselled according to PMTCT guidelines. In summary, women in our study belonged to five groups:

1. women who attended rural ANC clinics but were not counselled nor tested for HIV since such services were not available at these clinics

2. women who attended Mbale hospital but were not counselled nor tested for HIV

3. women who attended Mbale hospital and were counselled but not tested for HIV

4. women who attended Mbale hospital and were counselled and tested for HIV

5. women who were tested for HIV in non antenatal settings but did not receive the counselling as recommended by the PMTCT programme.

**Figure 1:**
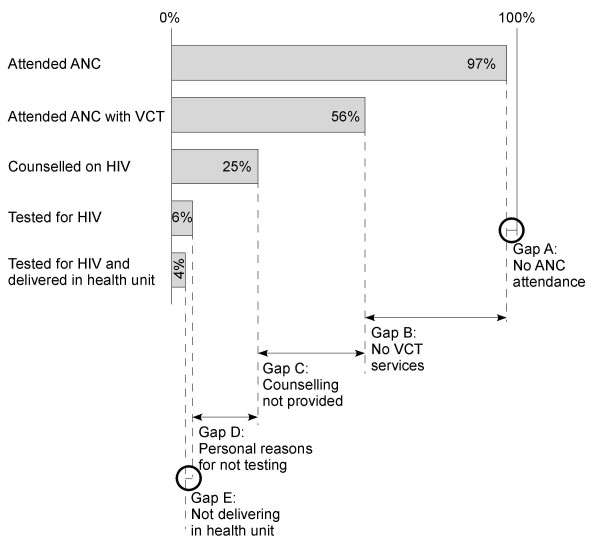
Gaps in implementation of the PMTCT programme among 457 women in Mbale, Uganda.

In our study almost all women (97%, 442/457) had attended an antenatal clinic but only 56% (256/457) had attended antenatal clinic with VCT services (Figure 1). Twenty five percent of the women (115/457) were counselled on HIV. Overall, 10% of the women (45/457) were tested for HIV. However, only 6% of the women (27/457) were tested for HIV in an antenatal setting, and the others (4%, 18/457) tested for HIV in a non-antenatal setting. Almost half of the women (47%, 216/457) delivered in a health facility, but very few (4%, 20/457) who had tested for HIV following antenatal VCT delivered in hospital (Table 2). Our study showed that only 4% of pregnant women fully utilized the PMTCT services in Mbale District.

**Table 2:** Logistic regression of factors influencing HIV testing among 115 women counselled on HIV, Mbale, Uganda

Variable	Tested n (%)	Did not test n (%)	Unadjusted OR (95% CI)	Adjusted OR (95% CI)
Residence				
Rural	18(22)	65(78)	1.0	
Urban	10(31)	22(69)	1.6(0.7 – 4.1)	

Age of mother				
15 – 24 years	15(27)	40(73)	1.0	
25 – 45 years	13(22)	47(78)	0.7(0.3 – 1.7)	

Education of mother				
0 – 7 years	16(21)	61(79)	1.0	
8 years or more	12(32)	26(68)	1.8(0.7 – 4.2)	

Occupation of mother				
Agriculture	23(23)	75(77)	1.0	
Other	5(29)	12(71)	1.4(0.4 – 4.3)	

Radio ownership				
No	4(15)	23(85)	1.0	
Yes	23(28)	58(72)	2.3(0.7 – 7.3)	

Religion of mother				
Muslim	16(24)	50(76)	1.0	
Christian	12(24)	37(76)	1.0(0.4 – 2.4)	

Marital status				
Married/cohabiting	24(24)	78(76)	1.0	
Single/widowed	4(31)	9(69)	1.4(0.4 – 5.1)	

Parity				
Multipara	16(18)	71(82)	1.0	
Primipara	12(43)	16(57)	3.3(1.3 – 8.4)	3.3(1.3 – 8.4)

Delivery in a health facility?				
No	8(14)	50(86)	1.0	
Yes	20(35)	37(65)	3.4(1.3 – 8.5)	

Who is your primary confidant?				
Another woman	17(22)	62(78)	1.0	
Husband	5(33)	10(67)	1.8(0.5 – 6.1)	

Age of husband				
18 – 24 years	2(17)	10(83)	1.0	
25 years or more	19(29)	46(71)	2.1(0.4–10.3)	

Education of husband				
0 – 7 years	11(24)	34(76)	1.0	
8 years or more	11(24)	34(76)	1.0(0.4 – 2.5)	

Do you fear to test for HIV?				
No	25(26)	72(74)	1.0	
Yes	3(17)	15(83)	0.6(0.2 – 2.2)	

Thus there was an accumulating dropout in the cascade from 3% of women who did not attend antenatal clinic up to the 93% who did not complete all the activities of PMTCT including antenatal voluntary counselling, antenatal HIV testing, and delivery in hospital to receive intrapartum and postnatal nevirapine. The gaps in PMTCT implementation were due to: no ANC attendance (Gap A); no VCT services (Gap B); counselling not provided even though VCT services were available (Gap C); personal reasons for not testing for HIV (Gap D); and not delivering in a health unit (Gap E) (Figure 1). Among women who were counselled on HIV, only 25% actually tested for HIV (Table 2).

### Barriers to PMTCT implementation

The most frequent reason for not testing for HIV was unavailability of VCT services (44%, Table 3). Due to lack of VCT services, the communities were not aware of the PMTCT programme and therefore did not encourage the women to test for HIV (Table 4). A health worker stated;

**Table 3:** Reasons given for no antenatal HIV testing among 457 women, Mbale, Uganda

Reason	Rural n (%)	Urban n (%)
No VCT services	155(85)	27(15)
Counselling or testing not offered	112(64)	62(36)
Fear of HIV testing	17(85)	3(15)
*Other	25(69)	11(31)

*Other included previous HIV testing, feeling in good health, and being away from home

**Table 4:** Reasons for no HIV testing among 457 women, Mbale, Uganda

**Reasons for no VCT services**
No VCT services except in Mbale Hospital
Community not sensitised about PMTCT


**Reasons for no counselling when VCT was available**
Shortage of staff
Poor motivation of staff
Shortages of materials (testing kits, drugs, health education)


**Reasons for no HIV testing after counselling**
Lack of privacy
Ineffective counselling
Fear
Need for further consultation
Perceived lack of benefits (antiretroviral drugs, infant milk)

"*Women do not test for HIV because their communities do not appreciate the value of VCT. Even if the woman is counselled, when she goes home for a second opinion, she will meet resistance from her circle*." (Jane, 57 yrs)

The second most frequent reason for not testing for HIV was lack of counselling (42%) even when VCT services were available in the health facility (Table 3). The health workers confirmed that counselling was fraught with many problems including availability and motivation of trained staff, availability of materials, and confidentiality (Table 4).

"*Trained counsellors are very few. To make matters worse even some of the trained counsellors are posted to work on other wards like surgical because of shortage of nurses*." (Henry, 48 yrs)

"*Sometimes the women may be willing to test for HIV but we have no testing kits and at times run short of nevirapine. Also we do not have videos for education of the women*" (Madina, 51 yrs)

*"Women fear that if they test in Mbale Hospital the other women waiting in the antenatal clinic will know that they tested for HIV and then start rumours" *(Jane, 57 yrs)

### Perceptions on HIV testing

Most women (82%) said they were not afraid of being tested for HIV if they were offered the opportunity. Overall, expressed fear contributed to only 5% of the reasons for no antenatal HIV testing. In contrast, health workers emphasised the importance of fear and reported that very few women accepted to test for HIV because the fear of knowing that they were HIV positive would cause them "to worry to death". In addition, women did not test because of the fear of other people knowing that they were HIV positive.

"*Many women believe that once you test HIV positive, you will worry to death. Women also think that once you test it is impossible to keep your results from your husband and other people. They fear beatings from husbands and gossiping by people*" (Namukose, 33 yrs)

### Consultation and confidentiality

Most women reported that their primary confidant was their mother followed by a sister or friend. The health workers confirmed that women often requested to go back home to seek advice before testing for HIV. Some women did not test for HIV because they feared that other women in the clinic would know they had tested by "*reading*" their faces and start rumours.

"*Women can sometimes fear to test because of other women in the clinic. You see the woman who wants to test has to go from the waiting room where all women gather and enter the Nurse's office. After testing she goes out of the Nurse's office in full view of the other women who will read on her face that she tested for HIV and begin rumours*" (Masete, 37 yrs)

### Perceived benefits of HIV testing

According to the health workers, women felt it was useless to test if they were not going to be treated in case they were HIV positive. In addition, having learnt that breastfeeding could cause HIV infection in the baby they felt that HIV positive women should be given milk for their babies, and thus did not agree to be tested since milk was not available at the health facility.

"Women ask 'How will my baby survive if I am dead?"

*"Women also say 'If a woman who is HIV positive can infect her baby (with HIV) by breastfeeding, then give us milk to feed the babies" *(Masete, 37 yrs)

### Factors associated with HIV testing

On logistic regression, being primipara (OR 2.6, 95% CI 1.2–5.8), urban dweller (OR 2.7, 95% CI 1.3–5.8), having been counselled on HIV (OR 6.2, 95% CI 2.9–13.2), and husband being the primary confidant (OR 2.3, 95% CI 1.0–5.5) were independently associated with HIV testing (Table 1). Among women who were counselled on HIV, primipara (OR 3.3, 95% CI 1.3–8.4) was associated with HIV testing (Table 2).

## Discussion

Our sample was similar to the population of Uganda except for religion. Since religion was not independently associated with antenatal HIV testing, we believe our findings can still be generalized to other parts of Uganda. Recall bias could have occurred, but is likely to have been minimal since most of the events women were asked to recall were one-time occurrences during the past one-year. The measurement of fear of HIV testing may have been subjective as reflected by the wide variation between the reports of the women and the health workers. Since our study was cross sectional, it was impossible to infer causality. Furthermore, variables such as perceptions on HIV may change over time and therefore perceptions held by the women at the time of the survey may have been different from their perceptions during pregnancy. Notwithstanding these limitations, we believe that our study has important findings for strengthening PMTCT implementation in Uganda.

Whilst most pregnant women (97%) attended antenatal clinic in government or private institutions, only 4% completed all the steps of the PMTCT programme. Previous studies have reported antenatal VCT acceptance rates of between 33% to 95%[4] in low income countries, indicating that the antenatal VCT acceptance in our study was very low. It was however similar to that reported in a national survey conducted by the Uganda AIDS Commission (13%) for 2003 but much lower than reported by Nakiwogga (38%) in Luwero District, Uganda in 2005[12]. Our interpretation is that the higher antenatal VCT acceptance rate in Nakiwogga's study was due to the longer period of implementation of the programme and increased antenatal VCT acceptance can be expected over time.

Our study strongly suggests that health system gaps were the major barriers to PMTCT implementation. The most frequent reason for not testing for HIV was unavailability of VCT services. Due to the lack of services, the communities were not aware of the PMTCT programme and therefore did not encourage women to test for HIV. While the Ministry of Health is responsible for implementing the policy in the regional (e.g. Mbale Hospital) and district hospitals, each district is responsible for implementing the policy in the health sub-districts, which include health centres and rural clinics. Local governments at the district level are supposed to provide leadership and oversight to community HIV/AIDS activities. New institutional structures such as the District AIDS Task Force (DAT) and the District HIV/AIDS Committee have been formed to enhance coordination of the multi-sectoral approach and resource mobilization. However, at the time of the survey these structures were not functional due to human and financial resource constraints[9]. Thus uptake of PMTCT, and in particular HIV testing, was greatly hampered by non-functioning HIV prevention structures in the districts. As has been demonstrated elsewhere, it is important to involve districts and communities in order to achieve wider PMTCT coverage[3,13,14].

The second most frequent reason for not testing for HIV was lack of counselling even when VCT services were available in the health facility. Consistent with other studies, the reasons for infrequent counselling in our study were unavailability and poor motivation of trained staff; unavailability of materials; and lack of confidentiality [15-21]. Counselling is key to the success of the PMTCT programme and the number and working conditions of counsellors in hospitals and health centres need further improvement. As has been shown elsewhere, counselling in hospitals and health centres can also be augmented by use of professional lay counsellors[13,14]. Furthermore, peer counsellors, including people living with HIV/AIDS, could be used to initiate counselling on HIV and to provide on-going support for women in the community[22,23]. Continuous supervision of the counselling is essential so as to maintain the necessary high quality[16,18,19,21]. Structural modifications in the clinic can improve on confidentiality and efforts should be made to ensure regular supply of materials, especially of the HIV testing kits.

In our study, we reported on the fears that women have regarding HIV testing including the fear of gossip, fear of worrying to death, and fear of beatings from husbands. Women perceive HIV testing as being synonymous with HIV/AIDS or having engaged in behaviours that could lead to HIV infection, conditions that are both stigmatised. We believe that the fears women have regarding HIV testing are a reflection of the stigma of HIV/AIDS. Stigma is described as "an attribute that is significantly discrediting"[24] and as "an attribute used to set the affected person or groups apart from the normalized social order, and this separation implies a devaluation"[25]. The discrediting nature of HIV/AIDS is reflected in the local names for AIDS such as *mpaawo atali kaaba *which means that "everybody will mourn"[26]. In HIV/AIDS, stigma may be linked to the fact that AIDS is a fatal and painful illness that ends in a miserable and undignified death[27,28], or to behaviours such as sexual promiscuity believed to lead to HIV/AIDS. In addition, the stigma related to HIV/AIDS is often superimposed on pre-existing stigmas of affected people[27]. Bharat et al[29] have identified three different types of HIV/AIDS stigma namely:

▪Self-stigma that manifests as self-blame and self-deprecation.

▪Perceived stigma that manifests in the fears that people have if they are HIV-positive and choose to disclose their HIV status to others.

▪Enacted stigma that occurs when people are actually discriminated against because they have, or are thought to have HIV.

Our study suggests that women in Mbale District have the perceived stigma of HIV/AIDS as described by Bharat et al[29]. The perceived stigma of HIV/AIDS is informed by enacted stigma in the society usually in the form of gossip, blame, avoidance of physical contact, discrimination, neglect and rejection. Furthermore, our study suggests that women are in a state of denial regarding their HIV status. Denial is the primary coping mechanism, and allows the women to remain in a state of ambiguity with regard to their HIV status[30]. Thus denial allows the women to maintain their status in society and "access to the humanizing benefit of free and unfettered social intercourse"[30]. Although our findings suggest that stigma was not a major barrier to PMTCT implementation in Mbale District, we hypothesise that as improvements are made in the delivery of antenatal VCT services, the personal barriers to PMTCT implementation including stigma will assume greater prominence. PMTCT services and antenatal VCT in particular, need to be integrated within preventive, educational, care and support initiatives for HIV through involvement of communities so as to address the fears and misconceptions that fuel stigma and discrimination[1,27].

Women felt a need to consult within their social environment before testing for HIV because of the social consequences of HIV testing[8,15,16,20]. Women were more likely to confide in other women rather than in their husbands. In a patriarchal society like that of Uganda, women are more likely to seek support and understanding from fellow women rather than from their husbands. However, women who said that their husbands were their primary confidants were more likely to test for HIV. In support of this, Bajunirwe et al[31] reported that the perception that the husband would approve of his wife's decision to test for HIV was the strongest predictor of whether the wife had the intention of testing or not. As other studies have shown, it is important to involve key people around the women and particularly the husbands in PMTCT so as to obtain their support for antenatal HIV testing[27,32].

Women do not see the reason to test for HIV if there are no tangible benefits for themselves and their infants. The women argue that it is not enough to protect the infant from HIV; women want to survive to raise their infants. Although these concerns are high on the priority list of the "3 by 5" initiative[33], these rural women may not benefit unless the PMTCT programme reaches them and those who test HIV positive are monitored and started on antiretroviral drugs as soon as they qualify[34]. The provision of infant formula has not been successful in the past because of the problems of affordability, feasibility, accessibility, and sustainability. Greater effort is needed in developing interventions that promote exclusive breastfeeding and which decrease the risk of HIV transmission[35]. It is important to do this in the whole community rather than in HIV positive women only so that exclusive breastfeeding is not perceived as an HIV preventive measure but rather as an intervention for the general promotion of child health[36]. Equally important is the establishment of follow-up programmes for HIV positive women and their infants for delivery of psychosocial support, prophylaxis for opportunistic infections, micronutrients and family planning services.

HIV testing was significantly lower in multipara women because they had a lower perceived risk of HIV. Multipara women have spent a longer period in their relationship and therefore feel secure and see no reason to test for HIV[37]. Conversely, multipara women have greater investment in the marital relationship and would stand to lose more if their partners abandoned them if they tested for HIV. Although the two reasons could co-exist, the fear of negative consequences is more plausible because of the low social position of women in rural Uganda[28].

HIV testing was significantly lower in rural women because they had poor access to antenatal VCT services, which were available only in Mbale Town. Our findings contrast with those of Bajunirwe et al[31] who found no differences between rural and urban women with regard to readiness to accept HIV testing in Mbarara District. The difference in the studies may be due to the fact that the rural clinics in Mbarara District were already implementing PMTCT whereas those in Mbale District were not. Rural and multipara women constitute a high-risk group for not testing for HIV and would benefit greatly from a rollout of the PMTCT programme into the rural clinics and from increased peer counselling in the community.

## Conclusion

In conclusion, the focus of the prevention of mother-to-child transmission of HIV needs to shift from the national Ministry of Health to the district and sub-district level and include more rural clinics in the programme; strengthen community mobilization particularly of rural communities; provide high quality services of antenatal voluntary counselling and HIV testing; develop and implement a programme of community based peer counselling on HIV; and provide follow up and care of HIV infected women and infants by provision of long-term antiretroviral drugs, counselling on infant feeding, psychosocial support, prophylaxis for opportunistic infections, and micronutrient supplementation. Given the high antenatal uptake, the potential for improving PMTCT coverage in Mbale District is high.

## Competing interests

The author(s) declare that they have no competing interests.

## Authors' contributions

CASK participated in the conception, design, and implementation of the study, statistical analysis, interpretation and drafting of manuscript. JKT participated in study conception, design and implementation of the study. TT participated in study conception, design, statistical analysis, interpretation, and drafting of manuscript. KH participated in study conception, design, interpretation and drafting of manuscript. All authors read and approved the final manuscript.

## Pre-publication history

The pre-publication history for this paper can be accessed here:


